# Larvicidal potential of *Trigonella foenum-graecum* and *Vitis vinifera* seed oil nanoemulsions against *Culex pipiens* (Diptera: Culicidae)

**DOI:** 10.1038/s41598-026-66903-5

**Published:** 2026-08-24

**Authors:** Manar M. Emara, Abla D. Abdel-Meguid, Nancy M. El-Shourbagy, M. S. Abdallah, Ahmed A. El-Sayed, Shaimaa Mahmoud Farag

**Affiliations:** 1 Entomology Department, Faculty of Science, Benha University, Benha, Egypt; 2 Photochemistry Department, Chemical Industries Research Institute, National Research Centre, Dokki, 12622 Giza Egypt; 3 Entomology Department, Faculty of Science, Ain Shams University, Abbasia, Cairo Egypt

**Keywords:** *Trigonella foenum-graecum*, *Vitis vinifera*, GC–MS, Nanoemulsion, *Culex pipiens*, Larvicidal activity, Biochemistry, Biological techniques, Biotechnology, Plant sciences

## Abstract

The increasing resistance of mosquito vectors to conventional insecticides highlights the urgent need for alternative and sustainable control strategies. Although plant-derived oils are selected due to their high content of bioactive compounds, especially unsaturated fatty acids and phenolic constituents, which have been reported to exhibit promising larvicidal activity and membrane-disrupting properties, their practical application is often limited by poor aqueous dispersibility, low physicochemical stability, and variable bioavailability. In addition, studies linking chemical composition to nano-enabled enhancement of larvicidal efficacy against *Culex pipiens* remain limited. Consequently, this study was established to develop nanoemulsions of *Trigonella foenum-graecum* (fenugreek seed oil) and *Vitis vinifera* seed oils (grape seed oils) and to evaluate their physicochemical characteristics and larvicidal activity. GC-MS analysis identified 14 and 34 compounds in fenugreek and grape seed oils, respectively, with fatty acids representing the dominant fraction. The prepared nanoemulsions exhibited nanoscale droplet sizes (194.8 nm for fenugreek and 55.21 nm for grape seed oil) and negative zeta potential values (-33.3 mV and − 29.9 mV), indicating good dispersion and colloidal stability. Transmission electron microscopy confirmed the formation of spherical and well-dispersed droplets. Larvicidal bioassays revealed a clear enhancement in toxicity for nanoemulsified formulations compared with crude oils. The fenugreek nanoemulsion showed the highest activity, with LC₅₀ values of 27.14 ppm and 23.20 ppm after 24 and 48 h of exposure, respectively. These findings indicate that nanoemulsification improves dispersion and enhances the biological performance of plant-derived oils. Overall, the results suggest that nanoemulsion-based formulations represent a promising approach for improving the efficacy of botanical larvicides. However, further studies are required to evaluate their performance under field conditions and to assess their environmental safety.

**Supplementary Information:**

The online version contains supplementary material available at 10.1038/s41598-026-66903-5.

## Introduction

Mosquito-borne diseases remain a major global public health concern, and vector control continues to represent one of the most effective strategies for reducing disease transmission^1,2^. Among mosquito species, *Culex pipiens* is of particular medical importance as a primary vector of West Nile virus, with a wide geographical distribution and strong ecological adaptability that enhances its epidemiological significance^3,4^.

Despite the long-standing reliance on synthetic insecticides, their intensive use has resulted in the rapid development of resistance in mosquito populations, thereby reducing control effectiveness^5,6^. Resistance mechanisms include target-site mutations, enhanced metabolic detoxification, and reduced cuticular penetration. In addition, the continued use of conventional insecticides raises concerns regarding environmental persistence and adverse effects on nontarget organisms^7,8^.

In response to these limitations, plant-derived oils have attracted increasing attention as environmentally compatible alternatives. Botanical insecticides are characterized by chemical diversity and multitarget modes of action, including membrane disruption, neurotoxicity, and induction of oxidative stress^7,9^. However, their direct application is often constrained by poor aqueous dispersibility, limited physicochemical stability, and variable bioavailability^10,11^.

Nanoemulsion-based delivery systems offer a promising approach to overcome these limitations. By improving the dispersion of hydrophobic compounds and increasing interfacial surface area, nanoemulsions enhance interaction with biological targets and improve formulation stability^12,13^. In larvicidal applications, reduced droplet size facilitates improved contact and potential penetration across larval barriers^14^.

Although considerable research has focused on nanoformulated botanical insecticides, most studies have reported essential oils rich in volatile terpenoids. In contrast, seed oils, which are predominantly composed of fatty acids and other lipophilic constituents, remain comparatively underexplored. Furthermore, integrated investigations linking chemical composition, physicochemical characterization, and larvicidal efficacy against *Culex pipiens* are still limited^15^.

Accordingly, the present study aimed to develop and characterize nanoemulsions of *Trigonella foenum-graecum* and *Vitis vinifera* seed oils and to evaluate their larvicidal activity against *Culex pipiens*. It was hypothesized that nanoemulsification would enhance dispersion, improve physicochemical stability, and increase larvicidal efficacy compared with crude oils.

## Materials and methods

### Insect rearing

Molting female *Culex pipiens* mosquito eggs were supplied by the Research and Training Center on Vectors of Diseases (RTC) located in the Faculty of Science at Ain Shams University. A regulated insectary environment at 27 ± 2 °C, 70 ± 10% relative humidity (RH), and a 14:10 (light: dark) photoperiod was maintained to sustain the colony for at least eight generations. In white enamel dishes measuring 35–40 cm in diameter and 10 cm deep, we placed the egg rafts. Each dish contained 1500 mL of distilled water. Larvae that had just hatched were dusted with powdered fish meal (TetraMin, Germany) twice a day^16^. To keep the distilled water from settling to the bottoms and sides of the pans or creating surface scum, it was stirred daily and replenished every two days. The breeding water was delicately aerated for about five minutes daily using a little air pump.

Regularly, we would collect the pupae, put them in plastic containers with distilled water, and then put them in screened wooden cages until they emerged as adults. The adults were housed in wooden cages of 24 × 24 × 24 cm and given cotton pads saturated with a 10% sucrose solution every day for four days. Next, every three days, the female mosquito were given a blood meal from the hamster rat so that they could lay their eggs. To improve the success rate of blood feeding, sucrose was removed 24 h before feeding.

Adult albino rats were obtained from the Animal House, Faculty of Science, Ain Shams University, Cairo, Egypt. All animals were housed under standard laboratory conditions with free access to food and water. Adults were given blood 48 h after being placed in oviposition containers with distilled water. Enamel bowls were used for the regular collection of egg rafts. Every day, the larvae were observed after being moved into new rearing pans measuring 25 × 30 × 15 cm that contained 3 L of tap water that had been aged^16,17^. Early third-instar larvae were used for bioassays.

### Plant oil procurement (cold-pressed oils)

Cold-pressed seed oils of *Trigonella foenum-graecum* and *Vitis vinifera* were obtained commercially and stored at 4 °C in dark glass containers to preserve their phytochemical stability, as recommended for plant-derived oils^18^.

#### Gas chromatography–mass spectrometry (GC-MS) analysis

The chemical composition of *T. foenum-graecum* and *V. vinifera* seed oils was determined using a gas chromatography–mass spectrometry (GC–MS) system (Shimadzu GC-MS-QP 2015 Plus, Kyoto, Japan). Chromatographic separation was performed using a capillary column (HP-type, 50 m × 0.2 mm internal diameter, 0.25 μm film thickness). Helium was used as the carrier gas at a constant flow rate of 1.0 mL/min. The injector temperature was maintained at 250 °C, and samples were introduced in split mode with a split ratio of 1:20. The oven temperature program was set as follows: initial temperature at 60 °C (held for 2 min), increased to 280 °C at a rate of 4 °C/min, and held at the final temperature for 10 min. The mass spectrometer was operated in electron ionization (EI) mode at 70 eV. The ion source temperature was maintained at 200 °C, while the interface temperature was set at 280 °C. Mass spectra were recorded over a scanning range of m/z 40**–**500. Identification of the separated compounds was achieved by comparing their mass spectra with those available in standard spectral libraries (e.g., NIST database)^19,20^, supported by retention time data where applicable. All analyses were conducted exclusively using GC**–**MS detection, and no flame ionization detector (FID) was employed in this study. This analytical approach enabled accurate separation and reliable identification of the chemical constituents present in the investigated seed oils.

### Preparation and optimization of nanoemulsions

Nanoemulsions (NEs) of *Trigonella foenum-graecum* and *Vitis vinifera* seed oils were prepared using the spontaneous emulsification method with slight modifications based on previously reported protocols^21,22^.

#### Selection of surfactant and cosurfactant

Tween 80 (polyoxyethylene sorbitan monooleate) was selected as a nonionic surfactant due to its high hydrophilic lipophilic balance (HLB = 15), which favors the formation of oil-in-water (O/W) nanoemulsions. Span 60 (sorbitan monostearate), with a low HLB value (HLB = 4.7), was used as a cosurfactant to reduce interfacial tension and enhance interfacial film flexibility. The combination of Tween 80 and Span 60 has been widely reported to improve nanoemulsion stability and reduce droplet size through synergistic effects.

#### Optimization of surfactant/cosurfactant ratio

Preliminary experiments were conducted to optimize the surfactant-to-cosurfactant ratio (Smix). Different weight ratios of Tween 80 to Span 60 (1:1, 2:1, 3:1, 4:1, and 5:1) were evaluated. The ratio of 5:1 (Smix) was selected as the optimal formulation, as it produced nanoemulsions with the smallest droplet size, lowest polydispersity index (PDI), and highest physical stability without phase separation during preliminary screening.

#### Preparation procedure

The oil phase consisted of either *T. foenum graecum* or *V. vinifera* seed oil (5% w/w). The surfactant mixture (Smix; Tween 80 to Span 60, 5:1) was added at 20% w/w. The remaining composition (75% w/w) was distilled water, forming an oil-in-water nanoemulsion system. Initially, the oil phase was mixed thoroughly with the Smix under magnetic stirring at 1000 rpm for 10 min at room temperature (25 ± 2 °C). Subsequently, the aqueous phase was added dropwise under continuous stirring to form a coarse emulsion. The resulting mixture was then subjected to high-energy ultrasonication using a probe sonicator (frequency: 20 kHz, power: 500 W) for 15 min in pulse mode (5 s on/5 seconds off) to avoid thermal degradation. The temperature was maintained below 30 °C during sonication using an ice bath.

#### Reproducibility and stability

All nanoemulsions were prepared in triplicate under identical conditions to ensure reproducibility. The prepared formulations were visually inspected for transparency, homogeneity, and absence of phase separation. Only stable nanoemulsions showing no signs of creaming, coalescence, or phase separation after 7 days of storage at room temperature were selected for further characterization and biological evaluation.

### Characterization of nanoemulsions

#### Thermodynamic stability studies

The thermodynamic stability of the prepared NEs was evaluated by subjecting them to various stress and temperature conditions. Samples were stored at 4 °C for three days and then at 45 °C for another three days; this cycle was repeated three times. The NEs were centrifuged at 2800 g for 30 min, followed by three freeze–thaw cycles (-20 °C for three days and then 25 °C for three days). Formulations were examined for signs of phase separation, creaming, or coalescence. Only NEs that passed all tests were selected for further characterization^12^.

#### Zeta potential and dynamic light scattering (DLS)

The zeta potential and distribution of particle sizes were assessed with a Nano-ZS particle size analyzer (Malvern Instruments Ltd., UK). A sonication period of 10–20 min had elapsed before the samples were measured^23^.

#### Transmission electron microscopy (TEM)

We used high-resolution transmission electron microscopy (HRTEM, JEOL JEM-2100) to examine the morphology and particle size of the prepared NEs. A 400-mesh carbon-covered copper grid was coated with a drop of the suspension, and the grid was left to air-dry at room temperature to prepare the samples^23^.

### Larvicidal bioassay

#### Stock preparation for larvicidal bioassay

The stock nanoemulsion was prepared directly from the optimized formulation and used as the primary dispersion for bioassay experiments. Based on the formulation composition, the stock solution contained 10% (v/v) active oil, corresponding to a concentration of 100,000 ppm. For larvicidal assays, serial dilutions were prepared from the stock nanoemulsion using distilled water to obtain the required test concentrations. All concentrations were expressed in ppm relative to the active oil content. Fresh dilutions were prepared prior to each experiment to ensure stability and accuracy. The prepared solutions were then used for larvicidal bioassays against third-instar larvae of *Culex pipiens* according to the WHO standard immersion method^24^. Groups of 25 larvae were transferred into 100 mL of test solution in disposable plastic cups under laboratory conditions (27 ± 2 °C, RH 70 ± 10%, 14:10 light–dark). Five concentrations of each oil and its NE were tested, each in triplicate, along with an untreated control. Mortality was recorded after 24 and 48 h.

### Statistical analysis

Data were analyzed using one-way ANOVA followed by LSD post-hoc multiple comparisons^25^ with SPSS version 26. Differences were considered significant at *P* < 0.05. Means were compared using Duncan’s multiple range test. Mortality percentages were expressed as mean ± SE (standard error). Data were subjected to probit analysis to determine LC_50_, LC_90_, slope, and χ^2^ values^26^.

## Results

### Identification of phytochemical compounds

#### GC–MS analysis of *Trigonella foenum-graecum* and *Vitis vinifera* seed oils

**Fig. 1:**
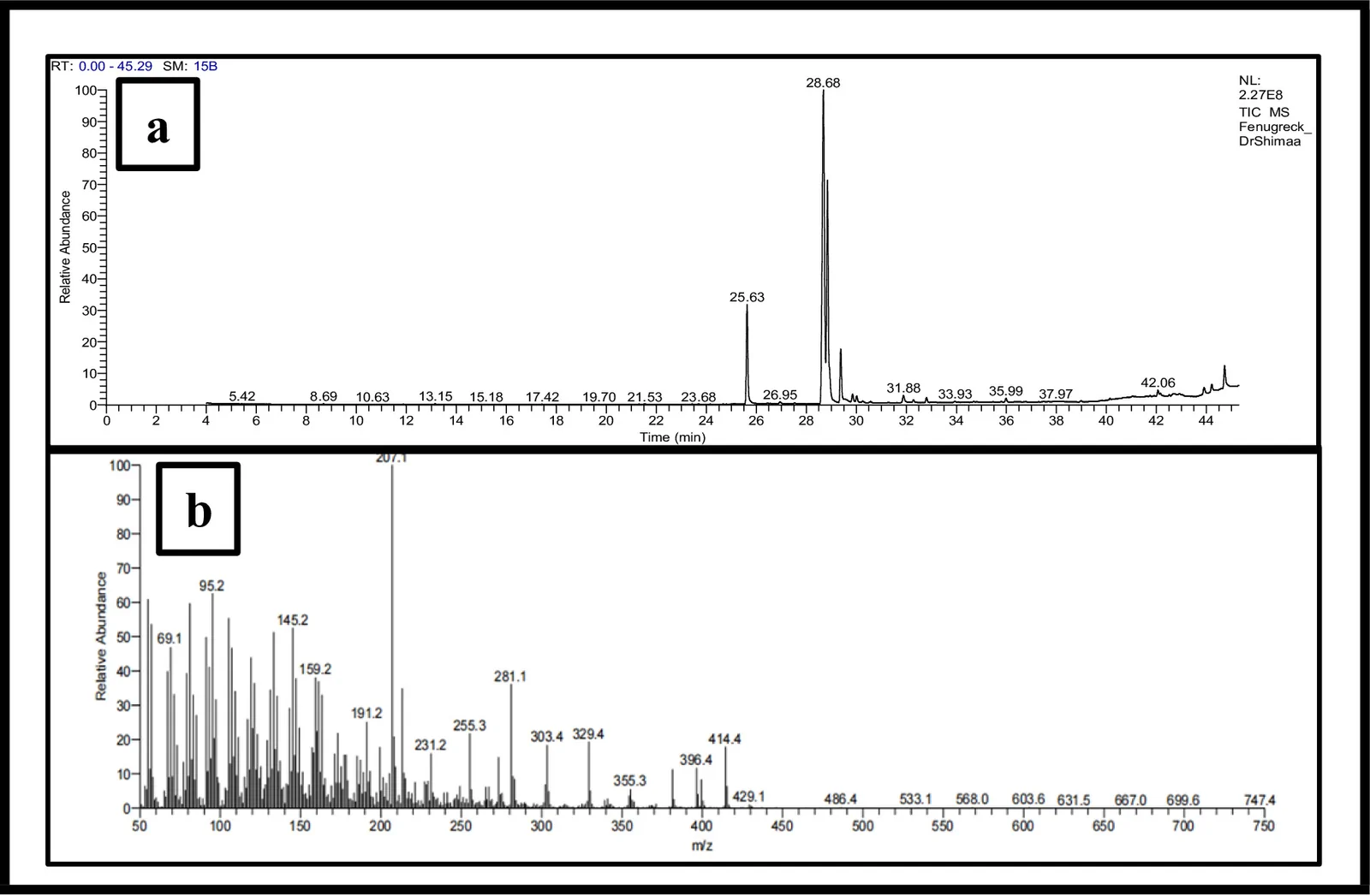
GC–MS chromatograms of (**a**) fenugreek seed oil and (**b**) grape seed oil showing the major peaks corresponding to their characteristic phytochemical constituents, including fatty acids, terpenes, and sterols.

GC–MS analysis identified the phytochemical constituents of *T. foenum-graecum* and *V. vinifera* seed oils based on retention time (RT), relative abundance (area%), molecular formula, molecular weight, and compound classification in Fig. 1a, b.

A total of 14 and 34 compounds were detected in fenugreek and grape seed oils, respectively, mainly belonging to fatty acids, alcohols, esters, terpenes, and sterols. Fatty acids represented the dominant fraction, accounting for approximately 94.05% in fenugreek oil and 79.17% in grape seed oil.

In fenugreek seed oil, the major constituents were linoleic acid methyl ester (46.1%), 11-octadecenoic acid methyl ester (26.7%), and palmitic acid methyl ester (10.57%). Similarly, grape seed oil was characterized by high proportions of linoleic acid methyl ester (58.65%), oleic acid methyl ester (19.33%), and palmitic acid methyl ester (9.41%). Minor components, including tocopherols and phytosterols (e.g., campesterol, stigmasterol, and β-sitosterol), were also detected in both oils at lower concentrations.

### Characterization of the prepared nanoemulsions

#### Thermodynamic stability

The thermodynamic stability of fenugreek and grape seed oil nanoemulsions was examined using a set of stress conditions, including centrifugation, freeze–thaw cycles, heating–cooling cycles, and dispersibility testing. During these evaluations, all samples were carefully observed for any visible changes such as turbidity, phase separation, creaming, coagulation, or cracking. No such changes were detected, indicating that the nanoemulsions maintained their physical integrity under the applied conditions.

#### Dynamic light scattering and zeta potential

**Fig. 2:**
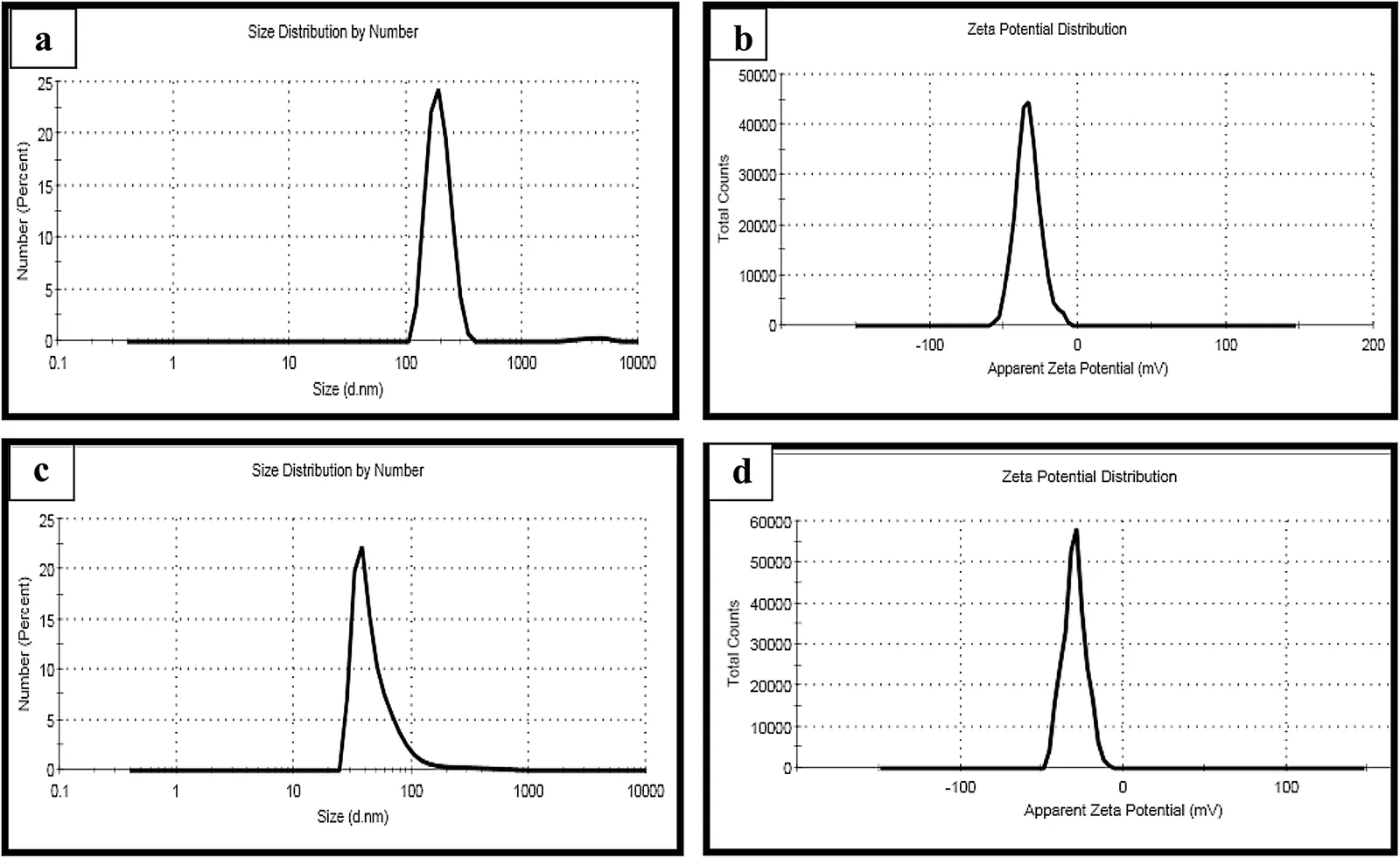
Physicochemical characterization of fenugreek seed oil nanoemulsion (FsO-NE) and grape seed oil nanoemulsion (GsO-NE). (**a**) Particle size distribution of FsO-NE obtained by dynamic light scattering (DLS), (**b**) zeta potential measurement of FsO-NE, (**c**) particle size distribution of GsO-NE, and (**d**) zeta potential measurement of GsO-NE.

The particle size distribution of the prepared nanoemulsions was analyzed using dynamic light scattering (DLS). The fenugreek nanoemulsion (FsO-NE) showed an average particle size of 194.8 nm, with a PDI value of 0.624 ± 0.056 (Table 1; Fig. 2a). This value reflects a moderately broad size distribution rather than a highly uniform system.

By comparison, the grape seed oil nanoemulsion (GsO-NE) exhibited a much smaller average particle size of 55.21 nm, with a PDI of 0.578 ± 0.032 (Table 1; Fig. 2c). Although narrower than FsO-NE, the distribution still falls within the range of moderate polydispersity. Regarding surface charge, the zeta potential of FsO-NE was measured at -3.3 mV, while GsO-NE showed a slightly lower value of -29.9 mV (Table 1; Fig. 2b, d).

**Table 1:** Particle size, polydispersity index (PDI), and zeta potential of fenugreek seed oil nanoemulsion (FsO-NE) and grape seed oil nanoemulsion (GsO-NE).

Formulation	Average particle size (nm)	PDI (± SD)	Zeta potential (mV)
FsO-NE	194.8	0.624 ± 0.056	−33.3
GsO-NE	55.21	0.578 ± 0.032	−29.9

#### TEM analysis

**Fig. 3:**
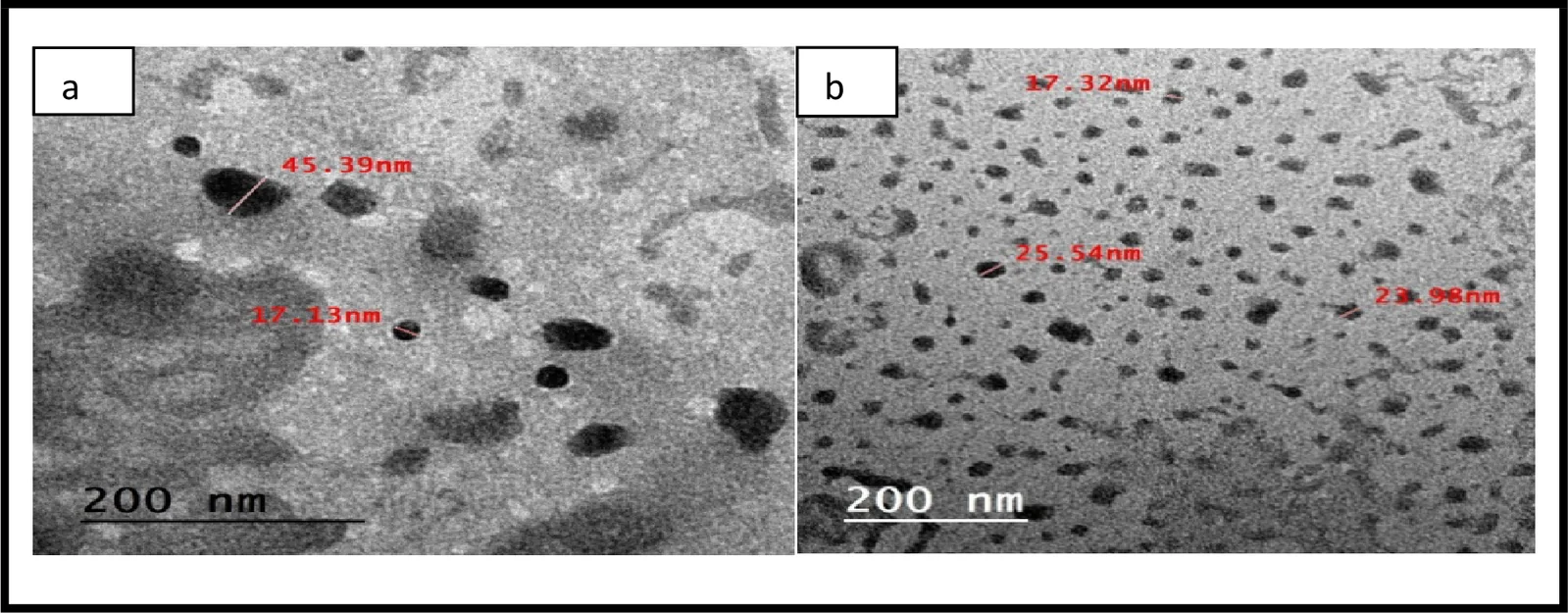
Transmission electron microscopy (TEM) micrographs of (**a**) fenugreek seed oil nanoemulsion (FsO-NE) and (**b**) grape seed oil nanoemulsion (GsO-NE) at a scale of 200 nm, illustrating the spherical morphology and nanoscale droplet distribution of both formulations.

Transmission electron microscopy (TEM) provided further insight into the morphology of the prepared nanoemulsions. The images confirmed that both systems were well dispersed in the aqueous medium, with no visible signs of droplet aggregation. The droplets appeared predominantly spherical and were distributed fairly evenly across the observed field. The particle size ranged between 17.13 and 45.39 nm for FsO-NE and between 25.54 and 37.32 nm for GsO-NE (Fig. 3a, b).

### Insecticidal activity

Fenugreek and grape seed oils, along with their corresponding nanoemulsions, were evaluated for their insecticidal activity against early larvae of *Culex pipiens* at different concentrations over 24 and 48 h.

#### Larvicidal activity of fenugreek seed oil and its nanoemulsion

The larvicidal activity of crude fenugreek seed oil and its nanoemulsion (FsO-NE) is presented in Table 2. Larval mortality increased progressively with increasing concentration and exposure time in both treatments. The highest concentration of crude oil (250 ppm) resulted in mortality rates of 90% and 94% after 24 and 48 h of exposure, respectively.

The nanoemulsion exhibited higher larvicidal activity at lower concentrations. Mortality reached 88%, 96%, and 100% at concentrations of 35, 40, and 45 ppm, respectively, after 48 h of exposure. No mortality was observed in the control group. Statistical analysis indicated significant differences among treatments (*P* < 0.05), confirming a concentration- and time-dependent response.

**Table 2:** Larvicidal activity of crude fenugreek seed oil and its nanoemulsion (FsO-NE) against *Culex pipiens* third-instar larvae after 24 and 48 h of exposure.

Treatment	Conc. (ppm)	24 h (% ± SE)	48 h (% ± SE)
Control	—	0.00 ± 0.00^a^	0.00 ± 0.00^a^
Crude oil	50	40.0 ± 0.33^b^	48.0 ± 0.33^b^
	100	48.0 ± 0.33^c^	63.2 ± 0.00^c^
	150	64.0 ± 0.00^d^	76.8 ± 0.57^d^
	200	72.0 ± 0.33^e^	80.0 ± 0.33^e^
	250	90.0 ± 0.00^f^	94.0 ± 0.33^f^
FsO-NE	25	41.2 ± 0.00^b^	60.0 ± 0.33^b^
	30	62.8 ± 0.33^c^	80.0 ± 0.33^c^
	35	76.0 ± 0.00^d^	88.0 ± 0.33^d^
	40	93.0 ± 0.33^e^	96.0 ± 0.33^e^
	45	97.0 ± 0.00^f^	100.0 ± 0.33^f^

Values are expressed as mean ± standard error (SE). Different superscript letters within the same column indicate significant differences (*P* < 0.05).

#### Larvicidal activity of grape seed oil and its nanoemulsion

The larvicidal activity of crude grape seed oil and its nanoemulsion (GsO-NE) is summarized in Table 3. Mortality increased significantly with both concentration and exposure time. The crude oil exhibited moderate activity, with mortality values of 36%, 48%, 72%, 80%, and 92% after 48 h at concentrations of 50, 100, 150, 200, and 250 ppm, respectively.

The nanoemulsion showed improved performance at lower concentrations. The highest concentrations (40 and 45 ppm) resulted in mortality rates of 80% and 92%, respectively, after 48 h. No mortality was recorded in the control group. Statistical analysis confirmed significant differences among treatments (*P* < 0.05).

**Table 3:** Larvicidal activity of crude grape seed oil and its nanoemulsion (GsO-NE) against *Culex pipiens* third-instar larvae after 24 and 48 h of exposure.

Treatment	Conc. (ppm)	24 h (% ± SE)	48 h (% ± SE)
Control	—	0.00 ± 0.00^a^	0.00 ± 0.00^a^
Crude oil	50	32.0 ± 0.33^b^	36.0 ± 0.00^b^
	100	45.6 ± 0.00^c^	48.0 ± 0.00^c^
	150	60.0 ± 0.00^d^	72.0 ± 0.00^d^
	200	76.8 ± 0.33^e^	80.0 ± 0.33^e^
	250	88.0 ± 0.33^f^	92.0 ± 0.33^f^
GsO-NE	25	32.0 ± 0.33^b^	36.0 ± 0.00^b^
	30	45.6 ± 0.33^c^	48.0 ± 0.33^c^
	35	60.0 ± 0.33^d^	72.0 ± 0.33^d^
	40	76.8 ± 0.00^e^	80.0 ± 0.67^e^
	45	88.0 ± 0.00^f^	92.0 ± 0.00^f^

Values are expressed as mean ± standard error (SE). Different superscript letters within the same column indicate significant differences (*P* < 0.05).

#### Relative efficiency of the tested compounds against *Culex pipiens*

As presented in Table 4, the LC_50_ values of fenugreek crude oil were 77.31 and 47.66 ppm after 24 and 48 h of exposure, respectively. In contrast, the fenugreek nanoemulsion exhibited markedly lower LC_50_ values (27.14 and 23.20 ppm, respectively) at the same exposure times.

Similarly, grape seed crude oil showed LC₅₀ values of 95.62 and 82.97 ppm after 24 and 48 h, respectively, whereas the corresponding nanoemulsion recorded significantly lower values of 30.64 and 29.15 ppm.

These results clearly indicate that nanoemulsification substantially enhances larvicidal efficacy. Based on LC_50_ values, the relative efficiency analysis demonstrated that the fenugreek nanoemulsion was approximately 2.84 and 2.05 times more effective than its crude counterpart after 24 and 48 h, respectively. Likewise, the grape seed oil nanoemulsion showed 3.12- and 3.84-fold higher efficacy compared to the crude oil at the same exposure times.

Overall, *Culex pipiens* larvae were more susceptible to nanoemulsified formulations than crude oils. Moreover, fenugreek oil exhibited higher larvicidal activity than grape seed oil under the tested conditions.

**Table 4:** Toxicity parameters (LC_50_, LC_95_), slope, χ², and relative efficiency of crude and nanoemulsified fenugreek and grape seed oils against *Culex pipiens* larvae after 24 and 48 h.

Compound	Formulation	Time (h)	LC_50_ (ppm) (LCL − UCL)	LC_95_ (ppm) (LCL–UCL)	Slope ± SE	χ²	Relative efficiency
Fenugreek oil	Crude	24	77.31 (60.23 − 97.79)	2201.34 (989.82–9772.60)	1.13 ± 0.18	3.36	1.00
		48	47.66 (35.47 − 59.19)	865.10 (501.91–2184.44)	1.31 ± 0.18	1.51	1.00
	Nanoemulsion	24	27.14 (25.63 − 28.37)	43.67 (41.01–47.71)	7.96 ± 0.80	2.70	2.84
		48	23.20 (20.87 − 24.88)	38.94 (36.55–42.80)	7.31 ± 0.92	1.08	2.05
Grape seed oil	Crude	24	95.62 (81.78 − 108.62)	533.91 (401.83–823.17)	2.20 ± 0.25	7.32	1.00
		48	82.97 (70.26 − 94.52)	418.43 (328.62–598.34)	2.34 ± 0.25	7.69	1.00
	Nanoemulsion	24	30.64 (29.04 − 32.03)	55.92 (50.67–64.85)	6.29 ± 0.69	1.98	3.12
		48	29.15 (27.55 − 30.49)	51.19 (47.05–57.94)	6.72 ± 0.71	2.67	3.84

LCL: lower confidence limit; UCL: upper confidence limit; χ²: Chi-square value; SE: standard error. Differences are considered statistically significant (*P* < 0.05).

## Discussion

### Identification of phytochemical compounds

#### GC–MS analysis of *Trigonella foenum-graecum* and *Vitis vinifera* seed oils

The predominance of fatty acids in both oils indicates a lipid-rich composition, which is commonly associated with biological activity in plant-derived insecticidal agents. Unsaturated fatty acids, particularly linoleic and oleic acid derivatives, have been reported to exert insecticidal effects through membrane disruption and oxidative stress induction^7^.

The presence of 11-octadecenoic acid derivatives may further contribute to toxicity by enhancing membrane permeability and facilitating the penetration of bioactive compounds into larval tissues^27^. Saturated fatty acids such as palmitic acid may also act synergistically with unsaturated components to improve overall efficacy^28^.

The detection of tocopherols and phytosterols also supports the biological potential of oils. Tocopherols function as natural antioxidants, contributing to the stability of lipid matrices, while phytosterols such as β-sitosterol have been reported to interfere with insect growth regulation and hormonal balance. However, these mechanisms were not directly investigated in the present study and should therefore be interpreted cautiously.

### Characterization of the prepared nanoemulsions

#### Thermodynamic stability

The stability observed under different stress conditions suggests that the prepared nanoemulsions are capable of maintaining their structural integrity, which is an important requirement for practical use. Similar findings have been reported for essential oil-based nanoemulsions, where the choice of surfactant system plays a key role in enhancing stability during storage and environmental exposure^13,15^.

#### Dynamic light scattering (DLS) and zeta potential

The PDI values obtained in this study (0.624 and 0.578) indicate moderately polydisperse systems rather than highly uniform dispersions. In general, PDI values below 0.3 are considered indicative of homogeneous nanoemulsions, whereas values between 0.3 and 0.7 reflect broader size distributions. Although the present formulations were not highly uniform, the obtained values remain within the acceptable range reported for many plant-based nanoemulsion systems. The negative zeta potential values measured for both formulations indicate the presence of electrostatic repulsion between droplets, which may contribute to colloidal stability. The FsO-NE formulation showed a value slightly exceeding the commonly referenced threshold of ± 30 mV, whereas the GsO-NE value was close to this threshold, indicating moderate rather than strong electrostatic stabilization. This interpretation is consistent with recent nanoemulsion studies^29^.

It is also worth noting that nanoemulsion stability is not determined solely by zeta potential. Other factors, such as the nature of the surfactant layer and steric effects, can play an important role in maintaining dispersion stability^11,12^.

#### TEM analysis

The smaller droplet size observed in GsO-NE suggests a finer dispersion system, which may increase the available surface area for interaction with the surrounding medium. In many nanoemulsion systems, particle sizes below 100 nm are often associated with improved stability and performance^14^.

The difference between particle sizes measured by TEM and DLS is expected, as TEM reflects dry-state dimensions, whereas DLS measures hydrodynamic diameter in a hydrated system^23^.

Overall, the nanoscale size and spherical shape of the droplets observed in both formulations are typical of oil-in-water nanoemulsions. Although GsO-NE showed a slightly narrower size range, both systems fall within the nanoscale region. While smaller particle sizes are often linked to better interaction with biological systems, such conclusions should be approached carefully, as biological activity depends on multiple factors beyond size alone^30^.

From a formulation standpoint, these results emphasize the importance of optimizing processing conditions to improve droplet uniformity and overall system stability. Further refinement of formulation variables, including surfactant selection and processing parameters, may help achieve more uniform nanoemulsion systems^10–12^.

### Insecticidal activity

#### Fenugreek seed oil and its nanoemulsion

The results of the present study indicate that *T. foenum-graecum* seed oil exhibits significant larvicidal activity against *Culex pipiens*, with mortality increasing progressively in relation to both concentration and exposure time. A markedly enhanced effect was observed following nanoemulsification, where the nanoemulsion achieved higher mortality at substantially lower concentrations than the crude oil. This improvement can be attributed to the physicochemical characteristics of nanoemulsions. The reduced droplet size provides a larger interfacial surface area, facilitating closer interaction with the larval cuticle and enhancing the delivery of lipophilic bioactive compounds into target tissues. In addition, improved dispersion in aqueous media promotes sustained contact between the formulation and the larvae, thereby increasing biological effectiveness^14^.

The larvicidal activity of fenugreek oil is likely associated with its phytochemical composition, particularly fatty acid derivatives and saponins. These compounds have been reported to disrupt membrane integrity, interfere with enzymatic activity, and impair larval metabolic processes^7^. Moreover, steroidal saponins may exert regulatory effects on insect growth and development, contributing to the overall toxicity profile^15^.

The enhanced efficacy observed for the nanoemulsion is consistent with previous studies demonstrating improved bioactivity of fenugreek-based nanoformulations. Such systems enhance the bioavailability and stability of active constituents, thereby increasing their interaction with biological systems^13^. Collectively, these findings indicate that nanoemulsification represents an effective strategy for improving the larvicidal performance of fenugreek oil.

#### Grape seed oil and its nanoemulsion

A comparable trend was observed for *V. vinifera* seed oil, where larvicidal activity increased in a concentration- and time-dependent manner. However, the crude oil exhibited lower efficacy compared to its nanoemulsion, which achieved similar mortality levels at reduced concentrations. The improved performance of the nanoemulsion can be explained by enhanced dispersion and increased surface area, which facilitate better interaction with larval surfaces and promote penetration of active compounds. These characteristics are widely recognized as key determinants of nanoemulsion efficiency in biological systems^13^.

The activity of grape seed oil may be attributed to its high content of unsaturated fatty acids, tocopherols, and phenolic compounds. These constituents are known to induce membrane disruption and oxidative stress, leading to impaired physiological functions in insect larvae^31^. The gradual increase in mortality over time suggests that the toxic effects involve both immediate structural damage and delayed biochemical responses.

Previous studies have reported similar findings, where nanoemulsified plant oils demonstrated enhanced larvicidal activity due to improved delivery and bioavailability of active compounds. For example, Vivekanandhan et al.^32^ showed that nanoemulsified formulations achieved higher toxicity against mosquito larvae compared to crude oils. Likewise, nanoemulsion systems have been reported to improve the persistence and effectiveness of botanical insecticides in aqueous environments^14^.

Although grape seed oil exhibited lower activity than fenugreek oil under the same conditions, its nanoemulsion showed a clear improvement in performance. This difference may be related to variations in phytochemical composition and the relative abundance of active constituents.

#### Relative efficiency and formulation impact

The comparative evaluation confirms that nanoemulsions are more effective than crude oils in reducing larval viability. The observed decrease in LC₅₀ values for nanoemulsified formulations indicates improved bioavailability and more efficient delivery of active compounds. Nanoemulsions facilitate the uniform distribution of hydrophobic substances in aqueous environments, enhancing their interaction with larval tissues and increasing uptake through the cuticle or digestive tract^30^. This improved delivery mechanism is a key factor underlying the enhanced larvicidal activity observed in the present study.

These findings are consistent with previous reports highlighting the superiority of nanoemulsion-based botanical insecticides over conventional formulations^13,14^. However, it should be noted that biological activity is influenced by multiple factors beyond droplet size alone, including formulation composition, surfactant properties, and overall system stability^11^.

Overall, the results support the potential application of nanoemulsion-based formulations as effective delivery systems for plant-derived larvicides, offering improved efficacy compared to crude oils.

## Conclusion

This study demonstrates that nanoemulsions formulated from *Trigonella foenum-graecum* and *Vitis vinifera* seed oils exhibit enhanced larvicidal activity against *Culex pipiens* compared to their corresponding crude oils, as indicated by lower LC₅₀ values and improved mortality responses. These findings highlight the potential of nanoemulsion-based systems to improve the efficacy of plant-derived larvicidal agents. However, the present results are limited to controlled laboratory conditions.

Further studies are required to evaluate field performance, environmental stability, and potential effects on nontarget aquatic organisms. In addition, investigation of the underlying mechanisms of larval mortality and optimization of formulation parameters will be essential to support practical application in mosquito control programs.

## Supplementary Information

Below is the link to the electronic supplementary material.

Supplementary Material 1

## Data Availability

All data generated or analysed during this study are included in the manuscript and its supplementary information files.
