# Supplementary material for: Larvicidal potential of Trigonella foenum-graecum and Vitis vinifera seed oil nanoemulsions against Culex pipiens (Diptera: Culicidae)
Source: Sci Rep. 2026 Aug 24;16:26481. doi: 10.1038/s41598-026-66903-5 (PMC13503835; doi:10.1038/s41598-026-66903-5)
Supplement: Supplementary file 1 [file 41598_2026_66903_MOESM1_ESM.docx]

**Larvicidal efficacy of *Trigonella foenum-graecum* and *Vitis vinifera* seed oil nanoemulsions against *Culex pipiens* (Diptera: Culicidae)**

Manar M. Emara¹, Ahmed A. El-Sayed^2*^, Shimaa M. Farag^3*^, Nancy M. El-Shourbagy¹, MS Abdallah¹, Abla D. Abdel-Meguid¹

^1^Entomology Department, Faculty of Science, Benha University, Benha, Egypt

^2^Photochemistry Department, Chemical Industries Research Institute, National Research Centre, Dokki, 12622 Giza, Egypt

^3^Entomology Department, Faculty of Science, Ain Shams University, Abbasia, Cairo, Egypt.

**Corresponding author**: Ahmed A. El-Sayed, E-mail: [ahmedcheme4@yahoo.com](mailto:ahmedcheme4@yahoo.com)

| Peak | RT | Area%  (Average rate) | Name | Molecular formula | Molecular weight (gm/mol) | | Compound nature |
| --- | --- | --- | --- | --- | --- | --- | --- |
| 1 | 25.62 | 10.57 | Palmitic acid methyl ester | C_17_H_34_O_2_ | 270 | Fatty acid | |
| 2 | 28.68 | 46.99 | Linoleic acid methyl ester | C_19_H_34_O_2_ | 294 | Fatty acid | |
| 3 | 28.85 | 26.9 | 11-Octadecenoic acid, methyl ester | C_19_H_36_O2 | 296 | Fatty acid | |
| 4 | 29.37 | 5.79 | Stearic acid Methyl Ester | C_19_H_38_O_2_ | 298 | Fatty acid | |
| 5 | 29.85 | 0.86 | Linoleic acid ethyl ester | C20H36O2 | 308 | Fatty acid | |
| 6 | 30.01 | 0.62 | 9-Octadecenoic acid (z)-, ethyl ester | C_20_H_38_O_2_ | 310 | Fatty acid | |
| 7 | 31.88 | 1.02 | 9-Octadecenoic acid, 12-hydroxy-, methyl ester, [R-(Z)] | C_19_H_36_O_3_ | 312 | Fatty acid | |
| 8 | 32.80 | 0.53 | Eicosanoic acid, methyl ester | C_21_H_42_O_2_ | 326 | Fatty acid | |
| 9 | 35.98 | 0.41 | Docosanoic acid, methyl ester | C_23_H_46_O_2_ | 354 | Fatty acid | |
| 10 | 42.06 | 0.63 | ç-Tocopherol | C_28_H_48_O_2_ | 416 | Alcohol | |
| 11 | 42.16 | 0.36 | 9,12,15-Octadecatrienoic acid, 2,3-bis[(trimethylsilyl)oxy]propyl ester, (z,z,z) | C_27_H_52_O_4_Si_2_ | 496 | Fatty acid | |
| 12 | 43.90 | 0.95 | Campesterol | C_28_H_48_O | 400 | Terpenes | |
| 13 | 44.21 | 1.08 | Stigmasterol | C_29_H_48_O | 412 | Terpenes | |
| 14 | 44.73 | 3.22 | á-Sitosterol | C_29_H_50_O | 414 | Terpenes | |

**Table S1**: Chemical characterization of fenugreek seed oil through GC-MS analysis.

**Table S1** summarizes the GC–MS analysis of *Trigonella foenum-graecum* (fenugreek) seed oil. The results indicate that the oil is predominantly composed of fatty acid esters, with linoleic acid methyl ester (46.99%) as the major constituent, followed by 11-octadecenoic acid methyl ester (26.9%) and palmitic acid methyl ester (10.57%). Smaller amounts of saturated and unsaturated fatty acid methyl and ethyl esters were also detected. In addition to fatty acids, minor bioactive components such as γ-tocopherol and phytosterols (campesterol, stigmasterol, and *β*-sitosterol) were identified. Overall, the profile indicates that fenugreek seed oil is rich in unsaturated fatty acids with the presence of antioxidant and sterol compounds that may contribute to its biological activity.

**Table S2**: Chemical characterization of grape seed oil through GC-MS analysis.

| Peak | RT | Area%  (Average rate) | Name | Molecular formula | Molecular weight (gm/mol) | Compound nature |
| --- | --- | --- | --- | --- | --- | --- |
| 1 | 25.62 | 3.66 | Hexadecanoic acid, methyl ester | C_17_H_34_O_2_ | 270 | Fatty acid |
| 2 | 28.62 | 16.98 | 9,12-Octadecadienoic acid (Z,Z)-, methyl ester | C_19_H_34_O_2_ | 294 | Fatty acid |
| 3 | 28.80 | 16.11 | 11-Octadecenoic acid, methyl ester | C_19_ H_36_O_2_ | 296 | Fatty acid |
| 4 | 29.37 | 5.53 | Octadecenoic acid, methyl ester | C_19_H_38_O_2_ | 298 | Fatty acid |
| 5 | 31.88 | 10.99 | 9-Octadecenoic acid, 12-hydroxy-, methyl ester, (Z)- | C_19_H_36_O_3_ | 312 | Fatty acid |
| 6 | 32.28 | 0.94 | 11-Eicosenoic acid, methyl ester | C_21_H_40_O_2_ | 324 | Fatty acid |
| 7 | 32.81 | 1.24 | DOCOSANOIC ACID, METHYL ESTER | C_23_H_46_O_2_ | 354 | Fatty acid |
| 8 | 35.79 | 0.50 | ISOCHIAPIN B | C_19_H_22_O_6_ | 346 | Fatty acid |
| 9 | 35.99 | 1.26 | Docosanoic acid, methyl ester | C_23_H_46_O_2_ | 354 | Fatty acid |
| 10 | 39 | 0.70 | 9,12,15-OCTADECATRIENOIC ACID | C_27_H_52_O_4_Si_2_ | 496 | Fatty acid |
| 11 | 40.04 | 0.88 | 03027205002 FLAVONE | C_27_H_30_O_15_ | 594 | Flavonoid |
| 12 | 40.14 | 1.98 | Ethyl iso-allocholate | C_26_H_44_O_5_ | 436 | Fatty acid |
| 13 | 40.69 | 0.70 | Oleic acid, 3-(octadecyloxy)propyl ester | C_39_H_76_O_3_ | 592 | Fatty acid |
| 14 | 41.06 | 1.64 | DOCOSANOIC ACID, 8,9,13-TRIHYDROXY-, METHYL ESTER | C_32_H_46_O_5_ | 402 | Fatty acid |
| 15 | 41.46 | 0.85 | Ethyl iso-allocholate | C_26_H_44_O_5_ | 436 | Fatty acid |
| 16 | 41.54 | 0.79 | DOCOSANOIC ACID, 8,9,13-TRIHYDROXY-, METHYL ESTER | C_23_H_46_O_5_ | 402 | Fatty acid |
| 17 | 41.75 | 1.13 | Ethyl iso-allocholate | C_26_H_44_O_5_ | 436 | Fatty acid |
| 18 | 41.93 | 0.64 | Ethyl iso-allocholate | C_26_H_44_O_5_ | 436 | Fatty acid |
| 19 | 42 | 0.35 | Ethyl iso-allocholate | C_26_H_44_O_5_ | 436 | Fatty acid |
| 20 | 42.16 | 2.41 | Ethyl iso-allocholate | C_26_H_44_O_5_ | 436 | Fatty acid |
| 21 | 42.52 | 1.89 | Ethyl iso-allocholate | C_26_H_44_O_5_ | 436 | Fatty acid |
| 22 | 42.62 | 1.00 | Ethyl iso-allocholate | C_26_H_44_O_5_ | 436 | Fatty acid |
| 23 | 42.72 | 0.51 | Oleic acid, eicosyl ester | C_38_H_74_O_2_ | 562 | Fatty acid |
| 24 | 42.92 | 0.92 | Ethyl iso-allocholate | C_26_H_44_O_5_ | 436 | Fatty acid |
| 25 | 43.04 | 1.24 | OLEIC ACID, 3-(OCTADECYLOXY)PROPYL ESTER | C_39_H_76_O_3_ | 592 | Fatty acid |
| 26 | 43.14 | 0.70 | DOCOSANOIC ACID, 8,9,13-TRIHYDROXY-, METHYL ESTER | C_23_H_46_O_5_ | 402 | Fatty acid |
| 27 | 43.87 | 4.15 | Betullin | C30H50O_2_ | 442 | Terpens |
| 28 | 44.07 | 1.76 | 9,12,15-OCTADECATRIENOIC ACID, 2,3-BIS[(TRIMETHYLSILYL)OXY ]PROPYL ESTER, (Z,Z,Z)- | C_27_H_52_O_4_Si_2_ | 496 | Fatty acid |
| 29 | 44.26 | 0.92 | DOCOSANOIC ACID, 8,9,13-TRIHYDROXY-, METHYL ESTER | C_23_H_46_O_5_ | 402 | Fatty acid |
| 30 | 44.31 | 1.03 | DOCOSANOIC ACID, 8,9,13-TRIHYDROXY-, METHYL ESTER | C_23_H_46_O_5_ | 402 | Fatty acid |
| 31 | 44.63 | 0.36 | Ethyl iso-allocholate | C_26_H_44_O_5_ | 436 | Fatty acid |
| 32 | 44.94 | 15.46 | à-Amyrin | C_30_H_50_O | 426 | Terpens |
| 33 | 45.09 | 0.22 | Betulin | C_30_H_50_O_2_ | 442 | Terpens |
| 34 | 45.15 | 0.53 | Ethyl iso-allocholate | C_26_H_44_O_5_ | 436 | Fatty acid |

**Table S2** presents the GC-MS characterization of grape seed oil, revealing that it is predominantly composed of fatty acid methyl esters. The major constituents include 9,12-octadecadienoic acid (linoleic acid) methyl ester (16.98%), 11-octadecenoic acid methyl ester (16.11%), and 9-octadecenoic acid, 12-hydroxy-, methyl ester (10.99%), indicating a high proportion of unsaturated fatty acids. Moderate amounts of hexadecanoic acid methyl ester (3.66%) and other long-chain fatty acid derivatives were also detected. Minor components such as flavone (0.88%) and several peaks corresponding to ethyl iso-allocholate were identified, suggesting the presence of bioactive secondary metabolites. Overall, the results confirm that grape seed oil is rich in unsaturated fatty acids with minor phenolic and sterol-related compounds that may contribute to its antioxidant and biological properties.
